# Quacks vs facts: Regulatory body discipline when clinicians spread COVID-19 mis/disinformation

**DOI:** 10.1177/17151635221076003

**Published:** 2022-02-07

**Authors:** Ai-Leng Foong-Reichert, Kelly A. Grindrod, Sherilyn K.D. Houle, Zubin Austin

**Affiliations:** From the University of Waterloo School of Pharmacy, Waterloo; From the University of Waterloo School of Pharmacy, Waterloo; From the University of Waterloo School of Pharmacy, Waterloo; Leslie Dan Faculty of Pharmacy, University of Toronto, Toronto, Ontario

The COVID-19 pandemic has undeniably changed pharmacy practice and ushered in an information overload that has brought the conversation about mis/disinformation in health care to the forefront (where *misinformation* is false information that was not intended to be harmful and where *disinformation* is false information that was intentionally created to cause harm).^1^ Combatting mis/disinformation and alternative-to-science views is not new, although it has unprecedented consequences in light of the pandemic. Historically, one of the reasons that professionals lobbied for legislation around regulation was to legitimize certain professions over others.^2,3^ They sought to marginalize “quacks and quackery” and alternative-to-science practitioners such as homeopaths, naturopaths and chiropractors and elevate science-based practitioners such as physicians, surgeons, dentists and pharmacists.^2^ When pharmacists first became a self-regulating profession in Quebec in 1870 and Ontario in 1871, regulation introduced entry-to-practice requirements that restricted access to the profession and protected the public by improving the quality of health care provided.^3^ Today, protection of the public continues to be the overriding goal of health professional regulators, although it is unclear how regulators are dealing with pharmacists who spread COVID-19-related mis/disinformation.

The consequences of the continued spread of COVID-19-related mis/disinformation are serious harm and death. According to the *Lancet* Commission on Vaccine Refusal, Acceptance and Demand, low vaccine uptake in certain US states has been fueled by politics and “systematic weaponised health communication” from anti-vaccine groups with a prominent social media presence.^4^ Low vaccine uptake rates will draw out the pandemic through continued transmission, possible resurgence of COVID-19 and emergence of variants and will ultimately “prolong the social and economic repercussions of the pandemic on families and communities.”^4^ Thus, it is of extreme importance that regulators take a clear stand against clinicians spreading mis/disinformation and that pharmacists and other clinicians combat mis/disinformation as described in a previous issue of this journal.^5^

At the time of writing, there have been no published disciplinary cases against Canadian pharmacists for behaviour related to COVID-19, but there are examples from other countries. Some cases involve intentional behaviour—a Utah pharmacist who fraudulently filled out COVID-19 proof-of-vaccination documents was fined and resigned their license to practice.^6^ Another Utah pharmacist illegally imported 550 kg of hydroxychloroquine and chloroquine through a non–FDA-approved manufacturer; the pharmacist was sentenced by the courts and is currently under probation with the regulatory body, with conditions on their license.^7-9^ In contrast, some pharmacists have been disciplined for the unintentional spread of disinformation—a New Zealand pharmacist unknowingly distributed a COVID-19 disinformation magazine after allowing the magazine to be left on the pharmacy counter along with other pamphlets; the pharmacist received a warning from the regulator.^10^ These examples illustrate that, on one hand, there is intentional, illegal behaviour, and on the other hand is the unintentional spread of disinformation. Our focus in this editorial is on the intentional spread of disinformation.

In the absence of published Canadian pharmacist cases, physician disciplinary cases provide examples of regulators’ responses to the spreading of disinformation. In March 2021, an Ontario pediatrician was disciplined for tweets claiming that lockdowns and vaccines were unneeded; the physician received a caution from the regulator.^11^ An Ontario family physician who has been spreading disinformation on social media has been banned from administering the COVID-19 vaccine, prescribing ivermectin and other medications not approved for COVID-19 and providing exemptions for COVID-19 testing, face masks or vaccination.^12^ According to the regulator’s disciplinary report, he is being further investigated for “making misleading, incorrect or inflammatory statements about vaccinations, treatments and public health measures for COVID-19” and for being “incompetent in relation to his communications.”^13^ But according to his tweets, he is unapologetic. Both of these Ontario physicians continue to influence their 46,000 and 124,000 Twitter followers, respectively.^12^ A physician from British Columbia who stated that vaccines are dangerous and that COVID-19 is not worse than the flu has been reprimanded and banned from speaking about COVID-19.^14^ The reprimand against this physician does not seem to be effective either—he is in a legal battle with the regulatory body regarding his right to free speech.^14^

In Canada, the right to freedom of expression is protected by the Charter of Rights and Freedoms. However, there is a difference between freedom of expression and spreading disinformation. In the disciplinary report available online, the College of Physicians and Surgeons of Ontario is clear that they are not curbing free speech or stifling criticism of public policy but that statements from a health professional, including social media posts, must be evidence-based.^15^ The onus is even greater when a health professional’s statements reach the public. For example, if a pharmacist posts an article containing misinformation on a pharmacist-only social media group to generate discussion, receive feedback and learn from their peers, this is likely appropriate given the context. However, if a pharmacist shares the same article on their personal social media profile or their pharmacy’s Facebook page, that would constitute spreading misinformation since they are acting as a health provider. In the cases of these physicians, the disinformation they shared is unproven and would be interpreted as medical advice by the general public since they present themselves online as physicians. In their ruling, the College highlighted that by not providing evidence to support their claims, “It would be expected and understandable if a certain proportion of the general public who read this statement decided to decline the vaccine with the assurance that they were acting on the guidance of a physician. For this reason, the Committee considers it irresponsible and a potential risk to public health, for the Respondent to have made this statement on social media in the middle of the pandemic.”^15^

Dr. Richard Friedman, a professor and physician, explains that any advice given by a physician, including any mis/disinformation created or shared, is a form of medical practice and should be subject to investigation and sanction by the regulator, including license revocation.^16^ He argues, “When doctors use the language and authority of their profession to promote false medical information, they are not simply expressing their own misguided opinions. Rather, they have crossed the line from free speech to medical practice—or, in this case, something akin to malpractice.”^16^ If a pharmacist believes and spreads mis/disinformation, they pose harm to the patient and to society and may be viewed by regulators as practising below the standard of clinical competence.

Clearly, physician regulators in Canada are identifying and disciplining rogue members, but in pharmacy, there are similar cases but no published disciplinary action. For example, an Alberta physician is being investigated by Alberta Health Services and the regulator for prescribing and treating patients with ivermectin.^17^ However, to our knowledge, the pharmacist who sourced the ivermectin from an agricultural store has not been investigated by the pharmacy regulator, and the news articles reporting on the physician do not mention consequences for the pharmacist. Although regulators have published statements or guidance documents denouncing the spread of mis/disinformation or involvement in certain unproven therapies (such as hydroxychloroquine and ivermectin), further action by pharmacy regulators is lacking.^18-20^

As a self-regulating profession, pharmacy regulators must act quickly to quash the spread of disinformation by pharmacists and must sanction decisively to denounce and deter this behaviour. Regulators have a responsibility to protect the public, but allowing rogue pharmacists to spread disinformation without consequences indeed has the potential to harm the public. The spreading of disinformation by pharmacists needs to be addressed by regulators in alignment with what we have seen for physicians. The public deserves no less.
